# Supplementary material for: Machine Learning and Artificial Intelligence in Intensive Care Medicine: Critical Recalibrations from Rule-Based Systems to Frontier Models
Source: J Clin Med. 2025 Jun 6;14(12):4026. doi: 10.3390/jcm14124026 (PMC12194786; doi:10.3390/jcm14124026)
Supplement: Supplementary file 1 [file jcm-14-04026-s001.zip › jcm-3642196-supplementary.pdf]

Supplementary Material

Supplementary Table S1: Key Technological Milestones in Biomedical AI/ML Applications

Description of the upper timeline depicting landmark developments in general biomedical applications.

Year	Milestone	Description	References
1950	Turing Test	Proposed the Turing Test for machine intelligence	Turing (1950), [113]
1956	Dartmouth Conference	Coined the term 'artificial intelligence'	McCarthy et al. (2006), [114]
1964	ELIZA	First NLP program simulating conversation	Weizenbaum (1966), [115]
1965	DENDRAL	Expert system for identifying organic molecules	Lindsay et al. (1993), [116]
1971	INTERNIST-1	Comprehensive diagnostic support system	Miller et al. (1982), [16]
1976	MYCIN	Expert system for infectious disease diagnosis	Shortliffe (1976), [117]
1983	Neocognitron	Hierarchical neural network for visual patterns	Fukushima (1980), [118]
1987	DXplain	Statistical clinical decision support system	Barnett et al. (1987), [21]
1998	ImageChecker	First FDA-approved CAD for mammography	Freer & Ulissey (2001), [119]
2000	SVMs in Genomics	Cancer classification using gene data	Brown et al. (2000), [120]

2007	Watson	IBM AI system for medical decision support	Ferrucci et al. (2010), [121]
2012	AlexNet	CNN that revolutionized medical image analysis	Krizhevsky et al. (2012), [122]
2016	IDx-DR	FDA-approved autonomous AI for diabetic retinopathy	Abràmoff et al. (2018), [123]
2022	AlphaFold2	AI for accurate protein structure prediction	Jumper et al. (2021), [124]
2025	AMIE	LLM-based AI with high diagnostic accuracy	Tu et al. (2025), [125]

Supplementary Table S2: Key Technological Milestones in AI/ML Applications for Intensive Care Medicine

Description of the lower timeline illustrating innovations specific to intensive care medicine.

Year	Milestone	Description	Reference Year
1972	HELP	First hospital information system with clinical decision support.	Warner, H. R. (1979). Computer-assisted medical decision-making. Academic Press. [126]
1981	APACHE	First severity-of-disease scoring system for ICU patients.	Knaus, W. A., et al. (1981). Critical Care Medicine, 9(8), 591-597. [127]
1993	VentPlan	AI-based system for mechanical ventilation management in ICU.	Rutledge, G. W., et al. (1993). Artificial Intelligence in Medicine, 5(1), 67-82. [128]
1996	MIMIC	First publicly available, large-scale ICU database for research.	Moody, G. B., & Mark, R. G. (1996). Computers in Cardiology, 657-660. [129]
2006	MIMIC-II	Enhanced ICU database with expanded physiological waveforms and clinical data.	Lee, J., et al. (2011). IEEE Engineering in Medicine and Biology Society, 8315-8318. [23]
2015	MIMIC-III	Comprehensive ICU database of 40,000 patients with de-identified clinical data.	Johnson, A. E. W., et al. (2016). Scientific Data, 3, 160035. [45]

2015	SICULA	Super ICU Learner Algorithm combining multiple models for mortality prediction.	Pirracchio, R., et al. (2015). The Lancet Respiratory Medicine, 3(1), 42-52. [24]
2015	TREWScore	Real-time early warning score for detecting sepsis with 85% accuracy.	Henry, K. E., et al. (2015). Science Translational Medicine, 7(299), 299ra122. [130]
2018	AI Clinician	Reinforcement learning system for sepsis treatment optimization.	Komorowski, M., et al. (2018). Nature Medicine, 24(11), 1716-1720. [27]
2021	MIMIC-IV	Latest version of MIMIC database with enhanced structure and coverage.	Johnson, A., et al. (2021). PhysioNet. [131]

Note. HELP – Health Evaluation through Logical Processing VentPlan; Ventilator Management Planning System (common expansion for VentPlan)
